# Spilanthol Inhibits Inflammatory Transcription Factors and iNOS Expression in Macrophages and Exerts Anti-inflammatory Effects in Dermatitis and Pancreatitis

**DOI:** 10.3390/ijms20174308

**Published:** 2019-09-03

**Authors:** Edina Bakondi, Salam Bhopen Singh, Zoltán Hajnády, Máté Nagy-Pénzes, Zsolt Regdon, Katalin Kovács, Csaba Hegedűs, Tamara Madácsy, József Maléth, Péter Hegyi, Máté Á. Demény, Tibor Nagy, Sándor Kéki, Éva Szabó, László Virág

**Affiliations:** 1 Department of Medical Chemistry, Faculty of Medicine, University of Debrecen, 4032 Debrecen, Hungary; 2 MTA-DE Cell Biology and Signaling Research Group, 4032 Debrecen, Hungary; 3 First Department of Medicine, University of Szeged, 6720 Szeged, Hungary; 4 HAS-USZ Momentum Epithel Cell Signalling and Secretion Research Group, 6720 Szeged, Hungary; 5 Department of Public Health, University of Szeged, 6720 Szeged, Hungary; 6 Institute for Translational Medicine, University of Pécs, Medical School, János Szentágothai Research Centre, 7624 Pécs, Hungary; 7 Momentum Gastroenterology Multidisciplinary Research Group, Hungarian Academy of Sciences, University of Szeged, 6720 Szeged, Hungary; 8 Department of Applied Chemistry, Faculty of Science and Technology, University of Debrecen, 4032 Debrecen, Hungary; 9 Department of Dermatology, Faculty of Medicine, University of Debrecen, 4032 Debrecen, Hungary

**Keywords:** dermatitis, pancreatitis, macrophage, nitric oxide, inducible nitric oxide synthase, spilanthol, NFκB, FOXO1, IRF

## Abstract

Activated macrophages upregulate inducible nitric oxide synthase (iNOS) leading to the profuse production of nitric oxide (NO) and, eventually, tissue damage. Using macrophage NO production as a biochemical marker of inflammation, we tested different parts (flower, leaf, and stem) of the medicinal plant, *Spilanthes acmella*. We found that extracts prepared from all three parts, especially the flowers, suppressed NO production in RAW macrophages in response to interferon-γ and lipopolysaccharide. Follow up experiments with selected bioactive molecules from the plant (α-amyrin, β-caryophylline, scopoletin, vanillic acid, trans-ferulic acid, and spilanthol) indicated that the N-alkamide, spilanthol, is responsible for the NO-suppressive effects and provides protection from NO-dependent cell death. Spilanthol reduced the expression of iNOS mRNA and protein and, as a possible underlying mechanism, inhibited the activation of several transcription factors (NFκB, ATF4, FOXO1, IRF1, ETS, and AP1) and sensitized cells to downregulation of Smad (TF array experiments). The iNOS inhibitory effect translated into an anti-inflammatory effect, as demonstrated in a phorbol 12-myristate 13-acetate-induced dermatitis and, to a smaller extent, in cerulein-induced pancreatitis. In summary, we demonstrate that spilanthol inhibits iNOS expression, NO production and suppresses inflammatory TFs. These events likely contribute to the observed anti-inflammatory actions of spilanthol in dermatitis and pancreatitis.

## 1. Introduction

Inflammation is the protective response of the innate immune system to an injury or pathogen invasion. First, pathogen or damage-associated molecular pattern (PAMP or DAMP) molecules liberated at the site of infection or tissue damage trigger pattern recognition receptors (PRRs). PRRs are expressed at high levels in myeloid cells, such as patrolling monocytes, tissue-resident macrophages, dendritic cells, and neutrophil granulocytes. Inflammatory macrophages release large amounts of proinflammatory cytokines, chemokines, and reactive oxygen and nitrogen species (ROS/RNS) locally, which kills and phagocytoses pathogens, activates the vascular endothelium, recruits and activates leukocytes [1]. Activated inflammatory macrophages play a central role in the orchestration of the inflammatory response. In addition to producing a wide variety of proinflammatory cytokines and chemokines, they are also important sources of reactive oxygen and nitrogen species (ROS/RNS). Macrophages are capable of a sustained, high-level release of nitric oxide (NO) because they upregulate inducible nitric oxide synthase (iNOS). The multifaceted role of NO, in both inflammation and the regulation of adaptive immune responses, is not fully understood [2]. The administration of NOS inhibitors in rodent models of inflammatory diseases curtails the severity of inflammation [3,4,5,6]. In addition, there is a correlation between the activity of the disease and iNOS expression in inflamed tissues or circulating monocytes [7]. NO mediates host defense and toxicity against microbes by inactivating enzymes via reactivity with thiol-groups and iron in iron-containing proteins [8]. NO can also combine with superoxide to form peroxynitrite, a highly reactive oxidant that attacks lipids and proteins. Peroxynitrite has microbicidal activity but can also inflict damage to the host tissue [9,10,11]. NO has a variety of effects on leukocyte function, including enhancement of macrophage motility, promotion of macrophage apoptosis in high concentrations, and modulation of cytokine synthesis. NO plays a role in resolving inflammation, along with TGF-β and IL-10 [12,13]. Therefore, NO is both a pro- and anti-inflammatory molecule and a measurable marker of active inflammatory processes. Inflammation becomes a major clinical challenge when maladaptive regulation leads to hyper-inflammation and sepsis, eventually leading to distant organ failure. Current clinical anti-inflammatory treatment is delivered largely through three classes of drugs: non-steroidal anti-inflammatory drugs (NSAIDs), corticosteroids and selective cyclooxygenase-2 inhibitors (COXIBs) [14]. Recent research findings also identified novel targets such as various secreted mediators or their receptors, intracellular signaling molecules, and transcription factors (NFκB/IκB, MAP kinases, and AP-1), enzymes (PLA2, iNOS) and the release of ROS/RNS [15]. Despite most modern approaches, such as in silico methods, rational design, and high-throughput screening, ethnopharmacology still constitutes a significant source of new drug candidates. The surging popularity of herbal medicines and a multi-target treatment approach to reduce doses and improve efficacy and safety, lend legitimacy to the search for naturally occurring complementary anti-inflammatory agents (e.g., curcumins, ginger, resveratrol, various plant polyphenols, cannabinoids, epigallocatechin, capsaicin, and berberin).

*Spilanthes acmella* (*S. acmella*), also known as *Acmella oleracea*, is a subtropical flowering herb from the Asteraceae family. *Acmella oleracea* has been used as a spice due to its acrid taste, the peculiar tingling palatal sensation, and the accentuating effect on the taste of other food ingredients [16]. The plant and its organic extracts also have a millennia-old history as a traditional folk remedy in many parts of the world [16]. From the Amazon basin to India, the flowers, leaves, and stem have been chewed to cure toothache, stomatitis, gingivitis, and sore throat due to a mild analgesic effect. In addition, *S. acmella* has been used as an antiseptic, antiviral, antiparasitic, and diuretic, and for wound healing [17]. A number of chemical compounds, including essential oils, phytosterols, lipophilic alkylamides, have been isolated from extracts of *S. acmella* [16].

In this study, we tested the anti-inflammatory properties of *S. acmella*. The effects of organic solvent plant extracts on RAW264.7 inflammatory macrophages were investigated. We find that the extracts reduce NO production in macrophages. Having tested six chemically pure substances selected from a compendium of reported bioactive molecules previously identified in *S. acmella* in the same assays, we attribute most of the observed effects to spilanthol, while trans-ferulic acid, vanillic acid, and scopoletin may also contribute to the inhibition of inflammatory macrophages. Down-regulation of iNOS decreases NO production, suggesting that spilanthol interfered with the gene expression of major inflammatory mediators. Spilanthol also conferred cytoprotection against the cell death of activated macrophages. Spilanthol was able to markedly relieve the symptoms and histological signs of acute inflammation in the mouse model of irritant contact dermatitis and reduced leukocyte migration in acute pancreatitis.

## 2. Results

**Figure 1:**
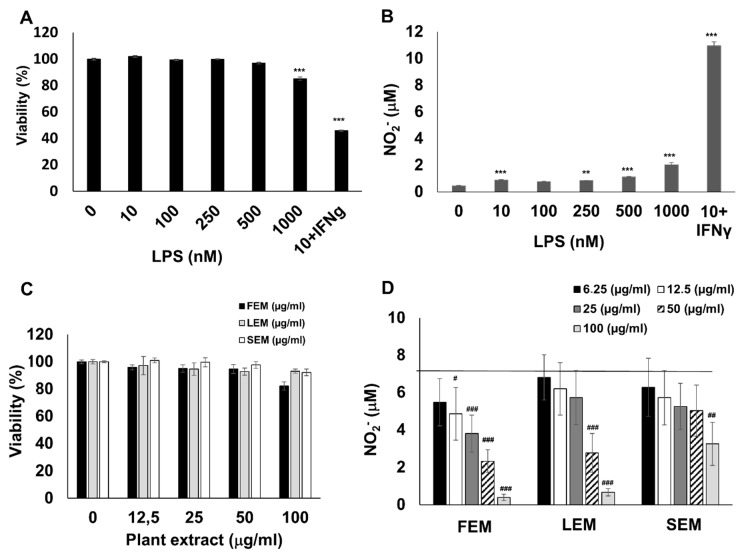
*S. acmella* extracts are non-toxic and suppress NO production in LPS + IFNγ-treated RAW264.7 macrophages. RAW cells were stimulated with the indicated concentrations of LPS with or without 10 ng/mL IFNγ. (**A**) Cell viability was measured with MTT assay and (**B**) NO release was assessed as NO_2_^−^ levels in the cell culture medium. (**C**) RAW264.7 cells were treated with (up to 100 µg extracted substance per ml of culture medium) the methanol extracts of floral (FEM), leaf (LEM) or stem (SEM) tissue from *S. acmella* and viability was measured with MTT assay. The extracts did not affect cell viability. (**D**) FEM, LEM, and SEM significantly and concentration-dependently decreased NO production. The solid horizontal line marks NO release from 10 ng/mL LPS + 10 ng/mL IFNγ-treated cells in the presence of no plant extract. # Hashmarks indicate significant (### *p* < 0.001) changes induced by IFNγ + LPS in viability (**A**) or NO production (**B**) as well as significant (# *p* < 0.05, ## *p* < 0.01, ### *p* < 0.001) inhibition of IFNγ + LPS-induced NO production by the plant extracts (**D**).

To obtain inflammatory macrophages, RAW264.7 cells were activated with lipopolysaccharide (LPS) and interferon-γ (IFNγ). In line with previous studies [18], the combination of LPS and IFNγ induced a massive production of nitric oxide, as indicated by increased nitrite content in the culture medium (Figure 1B). In addition, LPS + IFNγ treatment caused a 55% reduction in macrophage viability (Figure 1A). Pretreatment of cells with methanol extracts prepared from the flower (FEM), leaf (LEM), or stem (SEM) of *S. acmella* caused a concentration-dependent suppression of NO production without affecting cell viability (Figure 1C,D). These data confirmed that the extracts were non-toxic, and the decreased NO release was not due to altered cell viability.

**Figure 2:**
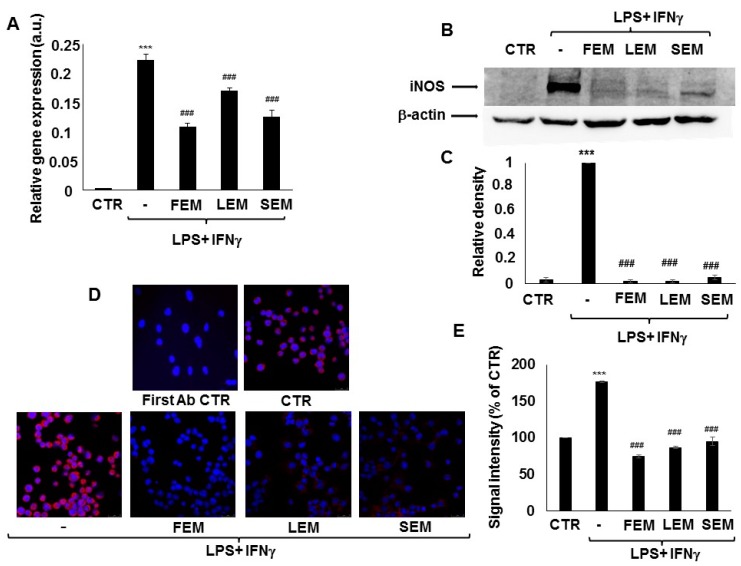
Flower (FEM), leaf (LEM), or stem (SEM) extracts of *S. acmella* mitigate expression of iNOS mRNA and protein in LPS + IFNγ-treated RAW264.7 macrophages. (**A**) RAW264.7 cells (CTR) upregulated relative gene expression of iNOS upon activation with 10 ng/mL LPS + 10 ng/mL IFNγ for 24 h in absence of plant extract (0). When the cells were cultured in the presence of FEM, LEM, or SEM, iNOS mRNA expression was reduced by 30–50%. (**B**) iNOS protein expression was also induced upon activation with 10 ng/mL LPS + 10 ng/mL IFNγ for 24 h in the absence of plant extract (lane 2 vs lane 1). FEM, SEM and LEM almost completely abrogated iNOS upregulation when the cells were cultured in the presence of plant extract. (**C**) Relative densities of the iNOS bands were determined. (**D**) Microscope images showing immunofluorescent staining of RAW264.7 macrophages for iNOS (red) with nuclear counterstain (blue). 10 ng/mL LPS + 10 ng/mL IFNγ treatment for 24 h in the absence of plant extract enhanced iNOS staining. FEM, SEM, and LEM extracts reduced the iNOS signal to 40–45% compared to LPS + IFNγ activated macrophages. (**E**) Intensities of the immunofluorescent iNOS signals were quantified in ImageJ software. (* Stars indicate significant (*** *p* < 0.001) induction of iNOS mRNA (A) or protein (C, E) production by IFNγ + LPS. #Hashmarks indicate significant (# *p* < 0.05, ## *p* < 0.01, ### *p* < 0.001) reduction of IFNγ + LPS-induced NO production by the plant extracts.).

In activated immune cells, upregulation of iNOS is responsible for most of NO production. We, therefore, checked if *S. acmella* extracts affected iNOS expression in the LPS + IFNγ treated RAW264.7 macrophages. LPS + IFNγ treatment was accompanied by a significant increase in iNOS mRNA levels within 24 h compared to the non-treated cells. Pretreatment with *S. acmella* LEM, SEM, or FEM extracts prevented the increased iNOS expression (Figure 2A). In accordance with these findings, western blotting showed increased iNOS protein in response to LPS + IFNγ activation, which was almost completely abrogated by LEM or FEM (Figure 2B,C). Data obtained from Western blot experiments were confirmed by immunofluorescent staining of immunostimulated RAW macrophages (Figure 2D,E).

**Figure 3:**
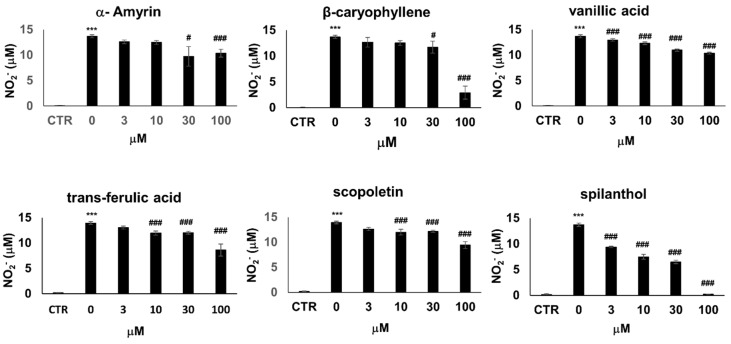
From a panel of selected chemical substances present in *S. acmella* extracts spilanthol is the most active in suppressing NO production. NO production of RAW264.7 cells activated with 10 ng/mL LPS + 10 ng/mL IFNγ for 24 h. Cells were pretreated with increasing concentrations (0–100 µM) of α-amyrin, β-caryophyllene, vanillic acid, trans-ferulic acid, scopoletin, and spilanthol. NO production was assessed from NO_2_^−^ levels present in the cell culture medium. (* Stars indicate significant (*** *p* < 0.001) induction of NO production by IFNγ + LPS. #Hashmarks indicate significant (# *p* < 0.05, ## *p* < 0.01, ### *p* < 0.001) reduction of IFNγ + LPS-induced NO production by the test compounds.).

A number of compounds have been isolated from the organic extracts of *S. acmella* and chemically characterized [16]. To identify the bioactive metabolite that provokes the iNOS inhibitory effects, we treated LPS + IFNγ activated RAW264.7 cells with six chemically pure bioactive substances present in *S. acmella*: spilanthol, β-caryophyllene, α-amyrin, trans-ferulic acid, vanillic acid, and scopoletin [19]. Treatment with trans-ferulic acid, vanillic acid, and scopoletin slightly decreased NO production (by up to 30%) (Figure 3) accompanied by a similarly mild reduction of cell viability (Figure S1). β-caryophyllene and α-amyrin decreased cell viability at and above 30 µM, thus the concomitant decrease of NO release most probably correlated with decreasing cell numbers. In the non-toxic concentration range, these substances did not affect NO levels. Lastly, spilanthol markedly suppressed NO production in a concentration-dependent manner without causing any toxicity (Figure 3, Figure S1). 

**Figure 4:**
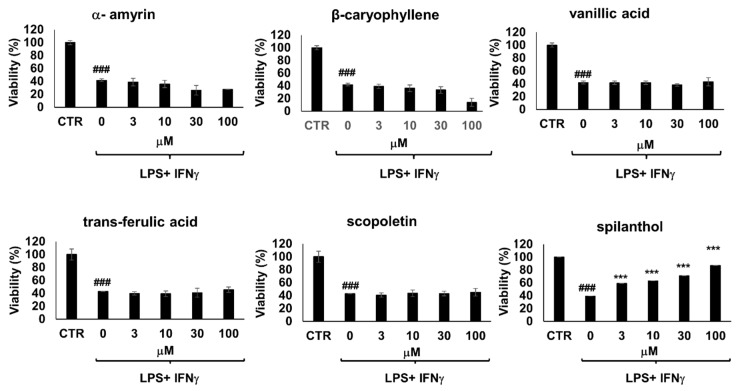
From a panel of selected chemical substances present in *S. acmella* extracts only spilanthol protects immunostimulated RAW macrophages from cell death. Cells were pretreated with the test compounds at the indicated concentrations for 2 h followed by treatment with LPS (10 ng/mL) and IFNγ (10 ng/mL) for 24 h. Cell viability was determined using MTT assay. (# Hashmarks indicate significant (### *p* < 0.001) decrease of viability caused by IFNγ + LPS treatment. *Stars indicate significant (*** *p* < 0.001) cytoprotection provided by spilanthol.).

Moreover, immunostimulated macrophages are known to develop cell dysfunction and display signs of cytotoxicity via iNOS-NO-ONOO^−^-DNA breakage-PARP1 activation pathway [18]. Of the 6 bioactive metabolites involved in our study, only spilanthol was able to prevent loss of viability in immunostimulated macrophages (Figure 4). The effect of spilanthol has also been confirmed using two additional assay methods, calcein and sulforhodamine assays (Figure S2). Therefore, next, we characterized the effect of spilanthol on iNOS expression.

**Figure 5:**
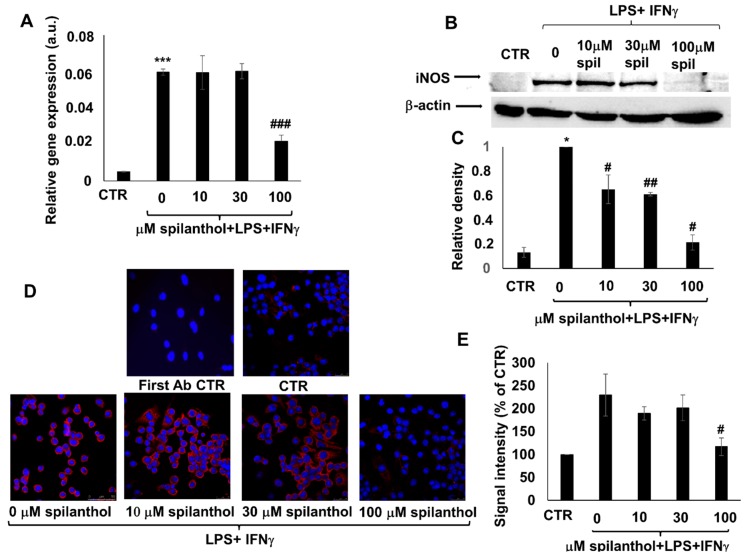
Spilanthol suppresses NO production in LPS/IFNγ-treated RAW264.7 macrophages by mitigating expression of iNOS mRNA and protein. (**A**) Increasing concentrations of spilanthol (0–100 µM) reduced iNOS mRNA expression in RAW264.7 macrophages activated with 10 nM LPS + 10 ng/mL IFNγ. (**B**) iNOS protein expression was also concentration-dependently inhibited by spilanthol. (**C**) Relative densities of the iNOS bands were determined. (**D**) Microscope images showing immunofluorescent staining of the spilanthol treated cells for iNOS (red) confirmed the gradual loss of iNOS staining with increasing concentrations of the drug. Blue is DAPI nuclear counterstain. (**E**) Intensities of the immunofluorescent iNOS signals were quantified in ImageJ software. (* *p* < 0.05, *** *p* < 0.001, # *p* < 0.05, ## *p* < 0.01, ### *p* < 0.001).

Pretreatment of immunostimulated macrophages with spilanthol reduced the level of iNOS mRNA (Figure 5A). Western blotting (Figure 5B,C) and immunofluorescent staining (Figure 5D,E) confirmed the inhibitory effect of 100 μM spilanthol on LPS + IFNγ-induced iNOS protein expression. 

**Figure 6:**
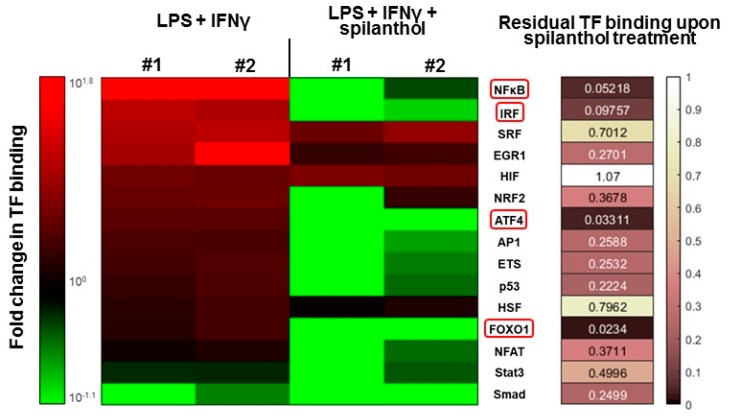
Spilanthol downregulates transcription factor pathways involved in inflammatory gene regulation. We tested the binding of 15 transcription factors to their cognate response elements in nuclear extracts of LPS + IFNγ and LPS + IFNγ + spilanthol treated cells by a transcription factor binding assay. The experiment was performed in duplicate (#1 and #2). The heatmap depicts the change of binding intensity of TFs compared to untreated cells. Binding comparable to that of the untreated cells is rendered in black, increased binding is shown in shades of red, and decreased binding in green, corresponding to the color bar. In the right panel, the level of residual TF binding after treatment with spilanthol is color-coded, so as darker colors denote increasingly pronounced inhibitory effect by spilanthol and white denotes no effect. We observed consistent downregulation in both experiments to ≤ 15% in the binding of NFκB, IRF, ATF4, and FOXO1.

Several transcription factors (e.g., NFκB, AP1, and IRF1) have been implicated in the transcriptional regulation of iNOS. Therefore, we investigated the transcription factor (TF) activation pathways that may be targeted by spilanthol in immunostimulated macrophages. Using a TF array, we found that several TFs become activated after LPS + IFNγ stimulation, with NFκB displaying the highest levels of activation (Figure 6). Treatment with 100 µM spilanthol nearly eliminated the binding of nuclear NFκB to its cognate response element, which is due to interference with upstream signaling, reduced translocation from the cytoplasm to the nucleus, or interference with consensus motif binding. Other TFs affected by spilanthol (> 85% inhibition) included ATF4, FOXO1, and IRF (Figure 6). TFs that were affected by spilanthol to a lesser degree (at least 60% inhibition) included AP1 and ETS. Smad was the only TF that was inhibited after LPS + IFNγ stimulation. Interestingly, spilanthol sensitized cells to Smad downregulation.

**Figure 7:**
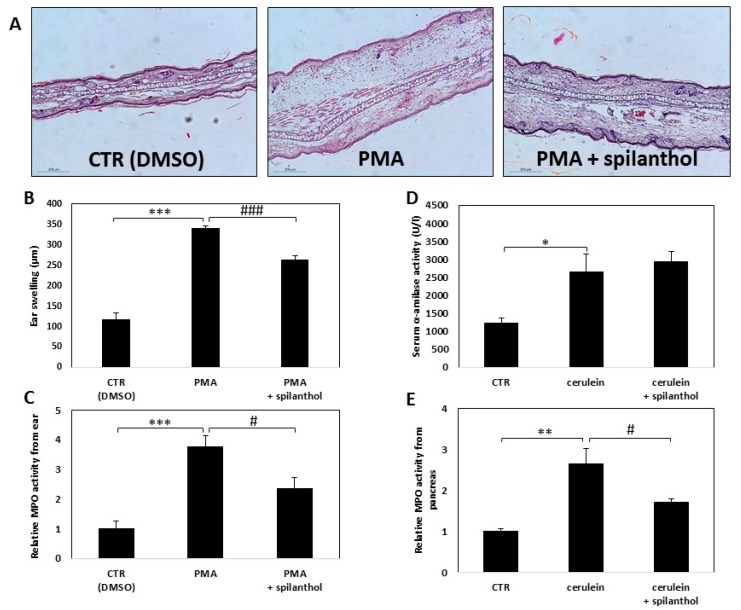
Spilanthol alleviates symptoms and decreases signs of inflammation in experimental dermatitis and reduces leukocyte infiltration in pancreatitis. (**A**) Dermatitis was induced as described in the Methods section. Micrographs of H&E stained ear sections from mice topically treated with vehicle, PMA, or PMA + spilanthol. (**B**) Spilanthol (10 µM solution, 10 μL applied topically on both sides of the ear) reduced the edematous response as demonstrated by decreased ear thickness. (**C**) Spilanthol reduces leukocyte infiltration into the inflamed ear, as shown by decreased MPO activity in the tissue. (**D**) Spilanthol (30 mg/kg) was ineffective in reducing pancreatic cell damage in the mouse cerulein-induced acute pancreatitis model, as indicated by serum α-amylase levels. (**E**) Spilanthol suppressed neutrophil infiltration into the pancreas as indicated by the decreased MPO level in the tissue. (* *p* < 0.05, ** *p* < 0.01, *** *p* < 0.001, # *p* < 0.05, ### *p* < 0.001).

In light of our finding that spilanthol inhibited NO production and perturbed inflammatory TF activation in macrophages, we next investigated the in vivo effectiveness of spilanthol in an irritant contact dermatitis model and in experimental acute pancreatitis. Skin irritation was provoked by smearing PMA onto the ears of BALB/c mice, control mice were treated with vehicle (DMSO) only. Erythema and swelling, indicating the development of inflammation, developed within 6 h after application of the irritant. To follow the inflammatory response, we measured the thickness of ears with a micrometer caliper. Hematoxylin-eosin stained histological sections of the ears revealed that PMA induced massive inflammation (Figure 7A). Spilanthol treatment resulted in a marked reduction of average ear thickness from 0.34 to 0.26 mm, indicating that spilanthol inhibited the edematous response (Figure 7A,B). The number of infiltrating leukocytes was also reduced markedly after spilanthol treatment (Figure 7C). 

Secretagogue overstimulation-induced pancreatitis was elicited by intraperitoneal injection of 50 μg/kg cerulein. Ensuing acinar cell death was indicated by elevated serum α-amylase levels (Figure 7D). Myeloperoxidase (MPO) activity, an indicator of neutrophil infiltration, increased substantially in the pancreatic tissue after cerulein treatment (Figure 7E). Intraperitoneal administration of 30 mg/kg spilanthol was ineffective in reducing pancreatic cell damage but inhibited neutrophil infiltration as indicated by the reduction of MPO activity in the excised tissue (Figure 7E).

## 3. Discussion

Billions of people in the world live outside the reach of modern medicine without quality health care systems. These people, particularly in rural, remote, and less developed areas, rely on traditional treatments when sick. The safety and benefit of this enormous number of consumers necessitate the rigorous, evidence-based scientific investigation into the pharmacological potency, efficacy, and pharmacodynamics of these traditional remedies. Folk remedies and herbal medicines are enjoying a revival and attract more interest from members of developed societies, thanks to their appeal to naturalist groups and the recognition by western medicine of the worth of traditional cures and procedures, as complementary medical practices [20,21]. The UN’s public health agency, the World Health Organization, embraced the potential of traditional medicine and the promotion of the safe and effective use of traditional medicine by researching and integrating its products into health systems in its Traditional Medicine Strategy 2014–2023.

*S. acmella* is a plant cultivated for its ornamental beauty, its use in cooking, and for the anecdotal curative powers in Indonesia, India, Bangladesh, Central-Africa, and northern Brazil. Various parts of the plant have long been known to alleviate painful maladies of the oral cavity and data in the literature also hint at its potential anti-inflammatory potential [17]. In the current study, we chose LPS-stimulated macrophages representing a central player in the inflammatory process and focused on NO production as a hallmark for inflammatory macrophage activation. Our findings showing that extracts prepared from various parts of the plant suppressed NO production in activated macrophages suggest the underlying mechanism for the potential anti-inflammatory effects of this plant. Different parts of the *S. acmella* plant had slightly different NO inhibitory effects, with flowers being the most potent and stems being the least potent. However, all three plant parts inhibited NO production significantly. As expected, *S. acmella* extracts suppressed iNOS expression both at the mRNA and protein levels, providing an explanation for the reduced NO production elicited by pretreatment with the extracts in LPS-induced macrophages.

*S. acmella* is a rich source of bioactive compounds, so we set out to identify the most likely candidate mediating inhibition of NO production. Several studies have been carried out to analyze the chemical composition of *S. acmella* extracts eventually yielding a large variety of compounds [17]. The most abundant class is N-alkylamides. Spilanthol, (2E, 6Z, 8E)-N_isobutylamide-2,6,8-decatrienamide, is the major representative of the alkylamides. Other bioactive components worth mentioning include triterpenoidal saponins [22], mono-, sesqui-, and triterpenoids (e.g., α-amyrin), phytosterols, coumarin derivatives (e.g., scopoletin), and lignocelluloid components (e.g., trans-ferulic acid and its derivative vanillic acid) [16,17]. 

Of the tested bioactive compounds of *S. acmella*, spilanthol clearly emerged as the most effective ingredient inhibiting NO production. Spilanthol was the only test compound that also prevented activated macrophage cell death caused by NO-derived peroxynitrite production, DNA breakage, and PARP1 activation. Spilanthol suppressed the levels of iNOS mRNA and protein suggesting that its effects are transcriptional. These data suggest that spilanthol may be responsible for the iNOS/NO suppressing effects of our plant extracts. Indeed, HPLC-UV analysis revealed a very high spilanthol content in the extracts with flower, leaf, and stem extracts containing 26.81 ± 0.04, 7.42 ± 0.04 and 3.59 ± 0.01% (wt/wt) spilanthol, respectively.

The exact mechanism by which spilanthol regulates iNOS and NO production is not yet known. Downstream signaling from LPS sensing TLR4 receptors engages the NFκB pathway, while IFN receptors mediate signals through the Jak/STAT1 pathway. Several interacting signaling pathways regulate iNOS expression and are dependent on the cell type and stimulus. 

Our TF array experiments highlight the central role of NFκB inhibition in spilanthol-treated cells. The NFκB pathway plays a pivotal role in the development of the inflammatory phenotype of various immune cells. As expected, LPS + IFNγ caused a marked (7–8 fold) increase in NFκB activation that was fully abolished by spilanthol. NFκB is thought to be predominantly responsible for elevated expression of iNOS and production of NO. The many direct and downstream target genes of NFκB include COX-2, TNFα, IL-6, and proIL1-β. Therefore, the NFκB suppressing effect of spilanthol likely decreases many inflammatory mediators in addition to iNOS.

AP-1 and interferon regulatory factor (IRF)-1 are also known to co-regulate the iNOS promoter [23]. These two TFs are also suppressed by spilanthol and may contribute to the effect on NO production. The transcription factor, Smad, was downregulated by LPS + IFNγ treatment and spilanthol sensitized cells to Smad suppression. Smad is the central transcription factor mediating the effects of TGFβ, a potent anti-inflammatory cytokine, which suppresses the expression of iNOS [24]. TGFβ–induced signals lead to reduced iNOS expression [25] via both transcriptional effects and destabilization of iNOS mRNA and protein [26]. There is a well-known antagonism between TGFβ and NFκB [27]. The RelA subunit of NFκB is responsible for the inhibition of TGFβ-induced phosphorylation, nuclear translocation, and DNA binding of SMAD signaling complexes induced by LPS, TNFα or IL1β. The mechanism of antagonism between these two signaling pathways relies on RelA-induced expression of inhibitory Smad7 protein [27], which antagonizes TGFβ signaling at various levels (for details see [28]). Whether spilanthol exerts NO inhibitory effects primarily via potentiation of Smad signaling or by inhibition of pro-inflammatory pathways is still in question. However, considering the mutual antagonism between these pathways, both options are theoretically possible. 

We chose a systemic and local inflammatory disorder in order to understand the effects of spilanthol on granulocyte migration. Pancreatitis seems to be one of the most suitable systemic inflammatory diseases for several reasons: (i) the incidence rate of pancreatitis continuously elevates causing an increasing healthcare need for specific treatment [29], (ii) the mortality rate is usually high due to the systemic inflammation [30], (iii) the mortality rate is unacceptably high (around 30%) in its severe form [30], (iv) granulocytes are involved in the pathomechanism of the disease [31], and finally but very importantly, (v) there is no specific treatment to cure acute pancreatitis [32]. The choice of dermatitis as a local inflammatory disease is justified by the very high incidence of the various forms of dermatitis (atopic, seborrheic, contact, irritative, radiation-induced, etc.), its effect on the quality of life and easily available animal model with local treatment option [33].

The iNOS inhibitory effect of spilanthol translated into anti-inflammatory actions as observed in the dermatitis model. The compound significantly reduced ear edema and granulocytic infiltration of the tissue. Reprogramming inflammatory transcription factor activation pathways are likely to be crucial as underlying mechanisms for this anti-inflammatory effect. As for the inhibition of granulocytic infiltration, spilanthol inhibits adhesion molecule expression [34,35] and similar events may contribute to reduced inflammatory cell migration in spilanthol treated mice. Interestingly, spilanthol failed to protect mice from secretagogue-induced acinar cell damage, although it significantly reduced granulocyte infiltration. This suggests that granulocyte infiltration is a secondary event triggered by acinar damage.

## 4. Materials and Methods 

### 4.1. Plant Collection and Preparation of Its Extracts 

Twenty grams (20 g) of dry powders of different parts of the plant (leaves, flowers, and stems) were extracted with 200 mL of methanol using Soxhlet extractor and dried under reduced pressure in a rotary evaporator (R-100; BÜCHI India Pvt. Ltd., Mumbai, Maharashtra, India). The resultant amorphous solid forms of the plant extracts were stored at 4 °C. 

The *Spilanthes acmella* plant was collected during the pre-monsoon period (March-May) from Doiwala, India (Latitude: 30°09′23.1″N; Longitude: 078°07′01.0″E) and was authenticated by the Botanical Survey of India (BSI), Dehradun (Acc. No. 118066). Different parts of the plant, including leaves, flowers, and stem (arial) parts without leaf and flower were separated, washed under tap water, and dried at 50 °C in a hot air oven. The dried samples were ground into fine homogenized powders using a sharp blender and stored in airtight bottles. Twenty grams (20 g) of dry powders were then extracted in 200 μL methanol using a Soxhlet extractor and dried under reduced pressure in a rotary evaporator (R-100; BÜCHI India Pvt. Ltd., Mumbai, Maharashtra, India). The resultant amorphous solid forms of the plant extracts were stored at 4 °C. 

### 4.2. HPLC-UV Method

HPLC-UV measurements were performed with a Waters 2695 Separations Module equipped with a thermostable autosampler (5 °C) and a column module (35 °C), a Waters 2996 Photodiode-array detector with a Waters Symmetry C18 column 3.9 × 150 mm, 5 µm) (each from Waters, Milford, MA, USA). Gradient elution at a flow rate of 1 mL/min was applied. Injection volume was 50 µL. The mobile phase A was water with 0.1% trifluoroacetic acid and B was acetonitrile with 0.1% trifluoroacetic acid. The gradient condition was as follows: initially 65% A and 35% B, 0–22 min linear change to 54% A and 46% B, 22–24 min linear change to 20% A and 80% B, and held to 34 min, 34–35 min linear change to 65% A and 35% B, and held to 45 min. The analytes were detected at the range of 210–400 nm, for qualitative analysis 230 nm was selected. The method was proved to be linear for spilanthol in the range of 3–50 µg/mL.

### 4.3. Cell Culture

RAW264.7 macrophages were cultured in a 5% CO2 incubator at 37 °C using an endotoxin-free modified Dulbecco’s minimal essential medium (Sigma–Aldrich, Budapest, Hungary) supplemented with 10% fetal bovine serum, 4 mM L-glutamine, and 10 μL per ml antibiotic, antimycotic solution (Sigma–Aldrich, Budapest, Hungary) 

### 4.4. Cell Treatments

Cells were seeded in 96 well tissue culture plates (10^4^/well) for cytotoxicity and total nitrite determination assays, 12 well tissue culture plates (10^5^/well) for real-time PCR measurements, 6 well tissue culture plates (10^6^/well) for Western blot analysis, and onto glass coverslips placed into 24 well tissue culture plates (2 × 10^4^/well) for immunofluorescence. The next day, cells were pretreated with either plant extracts or test compounds, including vanillic acid, α-amyrin, β-caryophyllene, trans-ferulic acid, scopoletin (Sigma-Aldrich, Budapest, Hungary), and spilanthol (Santa Cruz) at the indicated concentrations for 2 h followed by treatment with LPS (10 ng/mL) (Sigma–Aldrich, Budapest, Hungary) and IFNγ (10 ng/mL) (Bio-Vision) for 24 h. Test compounds were first dissolved in DMSO (stock solution) and were then further diluted in culture medium for cell-based experiments. DMSO concentrations were at or below 1% (v/v) in cell cultures which did not have any effect on the parameters measured.

### 4.5. Viability Assay

Cell viability was assessed using the colorimetric MTT assay [36]. After treatments, MTT (Sigma-Aldrich, Budapest, Hungary) was added to the cells (0.5 mg per mL) and incubated for an additional hour. The medium was then aspirated and the formazan crystals were dissolved by the addition of 100 μL dimethylsulfoxide. Optical density was determined at 540 nm in a plate reader (Tecan Spark, Tecan, Switzerland). Viability was calculated and expressed as a percent of control.

### 4.6. Sulphorhodamine B (SRB) Assay

Cells were fixed by the addition of 25μL 50% TCA to cells in 100 μL medium and incubated at 4 °C for 1 h. After washing three times with distilled water and air drying, 100 μL SRB solution (0.4% SRB/1% acetic acid) was added to the wells and plates were incubated for 10 min. After removal of the SRB solution, cells were washed five times with 1% acetic acid and air dried. Bound SRB was dissolved by adding 100 μL of 10 mM unbuffered Tris Base (pH 10.5) to each well and shaking for 30 min. Optical density was determined at 515 nm in a plate reader (Tecan Spark, Tecan, Switzerland). Viability was calculated and expressed as a percent of control.

### 4.7. CalceinAM Assay

Culture medium was aspirated and replaced by 50 μL CalceinAM solution (1 μM CalceinAM/medium). After 40 min incubation at 37 °C, fluorescent intensity was determined at 485/535 nm in a plate reader (Tecan Spark, Tecan, Switzerland). Viability was calculated and expressed as a percent of control.

### 4.8. Measurement of Total Nitrite Content

Nitrite content was measured using the Griess method as described [37]. Fifty μL of cell culture supernatants and sodium nitrite standards were transferred into a new 96 well plate. Another 50 μL of Griess reagent [50% of 1% sulfanilamide (Sigma–Aldrich, Budapest, Hungary) and 50% of 0.1% naphthylethylene-diamine dihydrochloride (Sigma–Aldrich, Budapest, Hungary) (dissolved in 5% H3PO4)] was added into each well and incubated at room temperature for 20 min. Absorbance was measured in a plate reader (Tecan Spark, Tecan, Switzerland) at 540 nm. 

### 4.9. RNA Extraction, Reverse Transcription, and Real-Time PCR Analysis

Total RNA was isolated from cells with TRI reagent (Molecular Research Center) and RNA purity was checked measuring A_260/280_ ratio. Reverse transcription and Quantitative real-time PCR was performed as described previously [38]. Gene expression levels were normalized to the geometric average of glyceraldehyde-3-phosphate dehydrogenase, β-actin, β-2-microglobulin, and hypoxanthine phosphoribosyltransferase-1 as reference housekeeping genes.

### 4.10. Immunofluorescent Detection of iNOS

Detection of iNOS was carried out by immunofluorescent staining following standard protocols [39] with modifications as follows. After treatment, cells were fixed in St. Mary solution (1% v/v acetic acid in ethanol) for 1 h at 4 °C, and then rehydrated in PBS for 5 min at room temperature. Coverslips were blocked with 1% BSA–20% FCS-Fc receptor blocker (kind gift from Dr. Zsolt Bacsó, Debrecen, Hungary) 1:400 diluted in phosphate-buffered saline (PBS)–Triton X-100 for 1 h at RT and then incubated overnight at 4 °C with monoclonal rabbit anti-iNOS antibody (Sigma–Aldrich, Budapest, Hungary) diluted 1:5000 in blocking solution. After 5 × 5 min washes in PBS, coverslips were incubated with AlexaFluor546-conjugated anti-rabbit IgG antibody (Life Technologies, Budapest, Hungary) diluted 1:500 for 1 h at room temperature. Nuclei were stained with 2μg/mL DAPI in PBS for 1 h at RT. Coverslips were mounted with Mowiol/ DABCO and samples were imaged with a Leica SP8 confocal microscope and evaluated with ImageJ software.

### 4.11. Western Blot Analysis

Western blots were carried out as previously described [40,41]. Briefly, cells were washed once in PBS and collected by scraping into 100 μL of ice-cold lysis buffer (62.5 mM Tris-HCl, pH 6.8, 2% SDS, 10% glycerol, 1 mM PMSF, and protease inhibitors). The extracts were further lysed with sonication and the supernatants were collected after centrifugation. Protein concentrations were determined with the Pierce BCA reagent (Thermo Fisher, Budapest, Hungary). Proteins (30 μg/lane) were separated in 10% SDS-PAGE and transferred to nitrocellulose membranes. Membranes were blocked with 5% non-fat dry milk in Tris-buffered saline (TBS) for 90 min. Primary antibodies against iNOS (rabbit monoclonal; Sigma-Aldrich, Budapest, Hungary) were applied overnight at 4 °C. After three washes in TBS containing 0.05% Tween 20, the secondary antibody (peroxidase-conjugated goat anti-rabbit IgG, Sigma-Aldrich, Budapest, Hungary) was applied for 1 h. Blots were washed in TBS containing 0.05% Tween 20 three times and once in TBS. After washing, the blots were incubated in Pierce Supersignal Chemiluminescent reagent (Thermo Fisher, Budapest, Hungary). For detection of luminescence, Chemidoc Touch gel documentation system (BioRad) was used and signals were quantified by densitometry using the ImageLab 6.0 software.

### 4.12. Transcription Factor Activation Profiling

Nuclear extract preparation and transcription factor activation profiling were carried out using the nuclear extract preparation Kit (Signosis, Santa Clara, USA) and the Oxidative Stress TF Activation Profiling Plate Array (Signosis, Santa Clara, USA) following the manufacturer’s instructions.

### 4.13. Contact Hypersensitivity (CHS)

Animal studies involving CHS experiments were approved by the institutional animal welfare committee (protocol 15/2016/DEMÁB) and were carried out as previously described [42] with modifications as follows. BALB/c mice were used, including 18 male and 18 female mice. All mice were 10–12 weeks old, and bred and maintained under temperature- humidity- and light-controlled conditions (21–23 °C, 30–60% relative humidity, 12 h light/dark cycle). All mice were fed ad-libitum VRF1 (P) food and water, and they were housed in Eurostandard Type II cages.

CHS was induced with phorbol 12-myristate 13-acetate (PMA). Animals were divided into three groups. The control group was treated with dimethylsulfoxide (DMSO), the vehicle of PMA. The PMA group was treated with 0.05% PMA and the PMA-spilanthol group with 0.05% PMA and 10 µM spilanthol (in DMSO). Before the induction of inflammation, ear-thickness of mice was measured with a Mitutoyo thickness gauge. Mice were then treated with DMSO or PMA (20 µL/ear applied with a micropipette, 10 µL to each side of the ear). Sixty minutes later the control and the PMA groups were treated with DMSO and the PMA-spilanthol group was treated with 10 µM spilanthol (20 μL smeared onto ears with micropipette, 10 µL to each side of the ear). The selection of the spilanthol concentration was based on experiments showing the effects of spilanthol on NO production (Figure 3). Six hours after the PMA treatment, the ear-thickness was measured again and mice were euthanized with isoflurane. The ears of the animals were removed for histology (hematoxylin-eosin staining) and MPO assays.

### 4.14. Cerulein-induced Acute Pancreatitis (AP)

Animal studies involving AP experiments were approved by the institutional animal welfare committee (protocol 25/2017/DEMÁB) and were conducted as previously described [43] with modifications as follows. Ten- to twelve-week-old male C57BL/6 mice were used for cerulein-induced AP. The animals were kept under standard conditions with a 12-h light/dark cycle. All mice were fed with a standard diet and received water ad libitum during the experiments. Mice (*n* = 42) were divided into three groups. In the cerulein-treated group, AP was induced by eight intraperitoneal injections of cerulein (50 μg/kg) at hourly intervals. In the cerulein + spilanthol group, mice received one intraperitoneal injection of spilanthol (30 mg/kg) 1 h before the first cerulein injection. (The DMSO content of the ca. 200 μL spilanthol solutions was 3.3%.) Mice in the control and cerulein groups were given saline (with 3.3% DMSO) only. Blood and pancreas were collected when mice were sacrificed 24 h after the first injection. The selection of the spilanthol dose was based on literature data showing that spilanthol has antinociceptive effect at this dose [44] and we hypothesized that anti-inflammatory effect may be observed in the same dose.

### 4.15. Serum Amylase Determination

Blood samples were taken by cardiac puncture and centrifuged at 5000 rpm for 15 min at 4 °C. Serum amylase activity was measured (using an enzymatic assay kit from Diagnosticum Zrt., Budapest, Hungary) in a kinetic reaction over 30 min using a Spark photometric plate reader (Tecan, Männedorf, Switzerland) at 405 nm.

### 4.16. Myeloperoxidase (MPO) Activity

Tissue MPO activity was measured as an indicator of neutrophil infiltration in the ear and pancreas as described [45] with modifications as follows. Tissue samples were weighed, thawed, and homogenized in 1 mL of 20 mmol/L phosphate buffer (pH 7.4), and then centrifuged at 13,000 rpm for 30 min at 4 °C. The resulting pellet was resuspended in 0.5 mL of 50 mmol/L phosphate buffer (pH 6.0) containing 0.5% HTAB (Sigma). The homogenates were then frozen in liquid nitrogen and thawed on three consecutive occasions before sonication. The samples were then centrifuged (13,000 rpm for 30 min at 4 °C), and the supernatants were collected for the MPO assay. Supernatant (100 µL) was mixed with 100 µL solution of 1.6 mM 3,3′,5,5′-tetramethylbenzidine (Sigma Aldrich, Budapest, Hungary) and 1 mM hydrogen peroxide. The mixture was incubated at 37 °C for 120 s, and the reaction was stopped with 200 μL 2 M H_2_SO_4_. The absorbance was measured at 450 nm and then normalized to tissue weight.

### 4.17. Statistical Analysis

All values are expressed as mean ± SEM and experiments were repeated at least three to four times. First, we ran Kolmogorov-Smirnov test to check if data have normal distribution. If they did we performed one-way ANOVA followed by Dunnett’s post hoc test. Data that didn’t show normal distribution were analyzed with Kruskal-Wallis tests followed by Dunn’s post hoc test. The value of *p* < 0.05 was considered significant.

## 5. Conclusions

Overall our study identified spilanthol as a potent bioactive molecule that reduces NO production in activated macrophages. Spilanthol interferes with activation of several TFs known to switch on the iNOS promoter. The iNOS suppressing effect of spilanthol translates into anti-inflammatory action (reduction of inflammatory cell migration) both in dermatitis and in pancreatitis. As a consequence, edema is reduced in dermatitis but acinar damage remained unaffected by spilanthol. Since extravasation of granulocytes and monocytes is a central and early event in most forms of inflammation, the beneficial effects of spilanthol may likely to be extended to a broad range of inflammatory conditions.

## Supplementary Materials

Supplementary materials can be found at https://www.mdpi.com/1422-0067/20/17/4308/s1. Figure S1. Cytotoxicity of selected chemical substances present in *S. acmella* extracts on RAW macrophages. Figure S2. Confirmation of the cytoprotective effect of spilanthol in RAW macrophages.

## Abbreviations

iNOS	inducible nitric oxide synthase
FEM	flower extract in methanol
LEM	leaf extract in methanol
SEM	stem extract in methanol
NFκB	nuclear factor kappa B
ATF4	activating transcription factor 4
FOXO1	forkhead box protein O1
IRF1	Interferon regulatory factor 1
ETS	E26 transformation-specific (transcription factor)
AP1	Activator protein 1
PMA	phorbol 12-myristate 13-acetate
PRR	pattern recognition receptors
ROS	reactive oxygen species
NO	nitric oxide
RNS	reactive nitrogen species
DAMP	damage associated molecular pattern
PAMP	pathogen-associated molecular pattern
TGFβ	transforming growth factor beta
IFNγ	interferon gamma
DABCO	1,4-diazabicyclo[2.2.2]octane
DAPI	4′,6-diamidino-2-phenylindole
PBS	phosphate buffered saline
TBS	Tris-buffered saline
LPS	lipopolysaccharide
NSAIDs	Non-steroidal anti-inflammatory drugs
COX-2	cyclooxygenase-2
COXIBs	cyclooxygenase-2 inhibitors
PLA_2_	phospholipase A_2_
CHS	contact hypersensitivity
DMSO	dimethylsulfoxide
HTAB	hexadecyltrimethylammonium bromide
MPO	myeloperoxidase
