# Supplementary material for: Spilanthol Inhibits Inflammatory Transcription Factors and iNOS Expression in Macrophages and Exerts Anti-inflammatory Effects in Dermatitis and Pancreatitis
Source: Int J Mol Sci. 2019 Sep 3;20(17):4308. doi: 10.3390/ijms20174308 (PMC6747447; doi:10.3390/ijms20174308)
Supplement: Supplementary file 1 [file ijms-20-04308-s001.pdf]

Supplementary material

Supplementary Figure S1

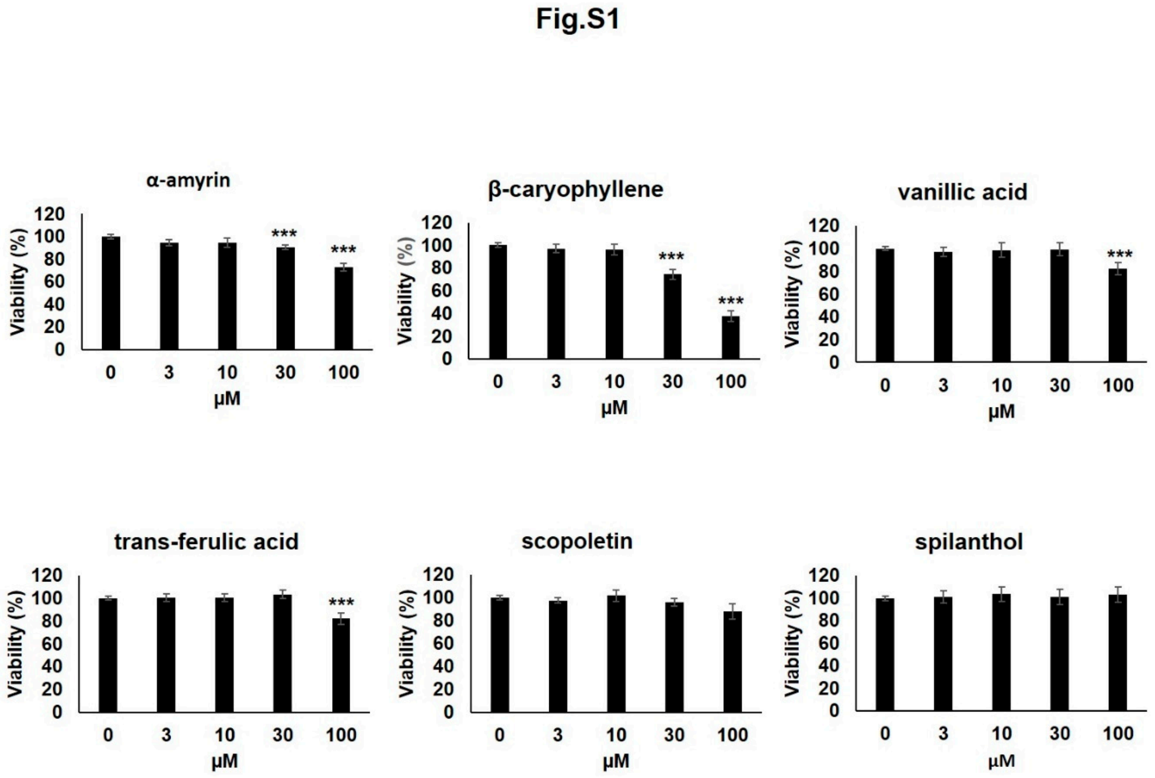

Figure S1. Cytotoxicity of selected chemical substances present in *S. acmella* extracts on RAW macrophages. Cells were treated with the test compounds at the indicated concentrations for 24 h, and cell viability was determined using MTT assay. (*Stars indicate significant (***$p < 0.001$) cytotoxic effect.)

Fig.S2

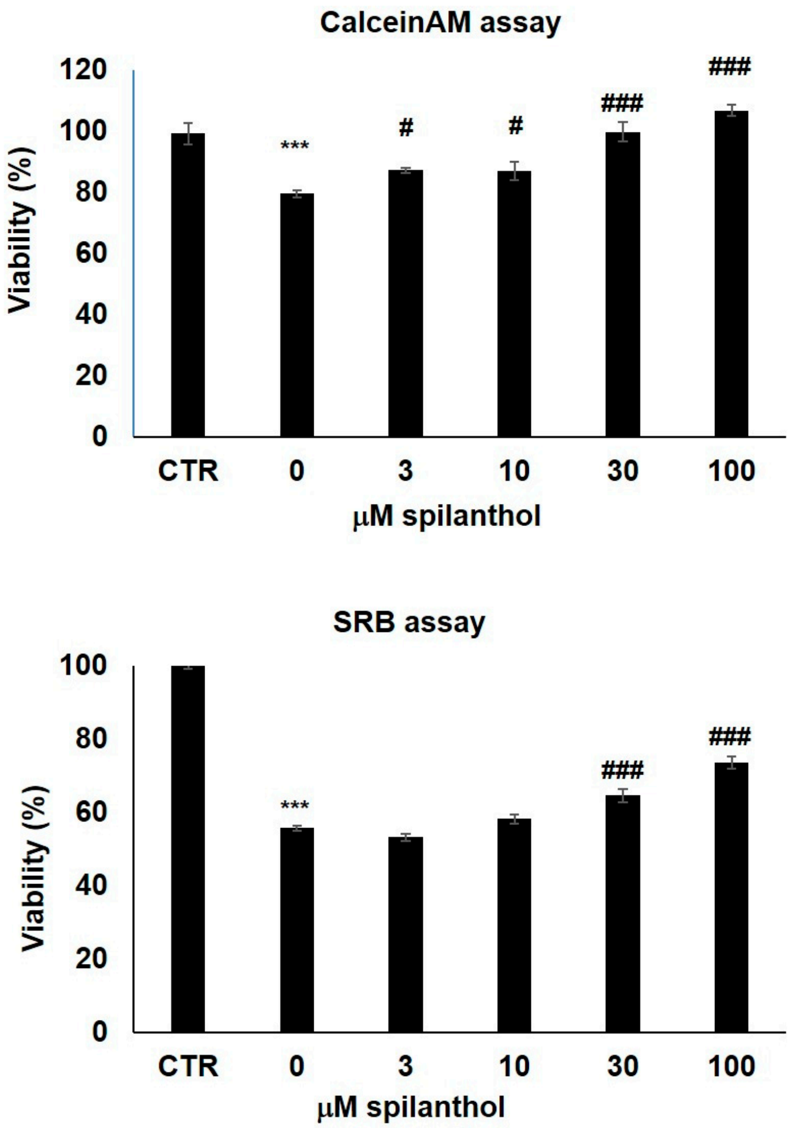

Figure S2. Confirmation of the cytoprotective effect of spilanthol in RAW macrophages. Cells were pretreated with spilanthol at the indicated concentrations for 2 h followed by treatment with LPS (10 ng/ml) and IFNγ (10 ng/ml) for 24 h. Cell viability was determined using (**A**) CalceinAM and (**B**) SRB assays. (*Stars indicate significant (***p < 0.001) decrease of viability caused by IFNγ + LPS treatment. #Hashmarks indicate significant (###p < 0.001) cytoprotection provided by spilanthol.).
